# ZhiJingSan Inhibits Osteoclastogenesis via Regulating RANKL/NF-κB Signaling Pathway and Ameliorates Bone Erosion in Collagen-Induced Mouse Arthritis

**DOI:** 10.3389/fphar.2021.693777

**Published:** 2021-05-28

**Authors:** Yuanyuan Ling, Jie Yang, Di Hua, Dawei Wang, Chenglei Zhao, Ling Weng, Dandan Yue, Xueting Cai, Qinghai Meng, Jiao Chen, Xiaoyan Sun, Weikang Kong, Lizhong Zhu, Peng Cao, Chunping Hu

**Affiliations:** ^1^ Affiliated Hospital of Integrated Traditional Chinese and Western Medicine, Nanjing University of Chinese Medicine, Nanjing, China; ^2^ School of Pharmacy, Nanjing University of Chinese Medicine, Nanjing, China

**Keywords:** ZhiJingSan, rheumatoid arthritis, bone erosion, osteoclasts, RANKL, NF-κB

## Abstract

Bone erosion is the most evident pathological condition of rheumatoid arthritis (RA), which is the main cause of joint deformities and disability in RA patients. At present, the conventional RA drugs have not achieved satisfactory effect in improving bone erosion. ZhiJingSan (ZJS), which is a traditional Chinese prescription composed of scolopendra (dried body of *Scolopendra subspinipes mutilans L. Koch*, *scolopendridae*) and scorpion (dried body of *Buthus martensii Karsch, Buthus*), exhibits anti-rheumatism, analgesic and joint deformities improvement effects. This study aimed to assess the therapeutic effect of ZJS on RA bone erosion and to elucidate the underlying mechanism. The effect of ZJS on RA bone erosion was investigated in a murine model of bovine collagen-induced arthritis (CIA), and the underlying mechanism was investigated *in vitro* in an osteoclast differentiation cell model. Administration of ZJS delayed the onset of arthritis, alleviated joint inflammation, and attenuated bone erosion in the CIA mice. Meanwhile, ZJS decreased the serum levels of TNF-α, IL-6, and anti-bovine collagen II-specific antibodies. Furthermore, ZJS treatment reduced the number of osteoclasts and the expression of cathepsin K in the ankle joints of CIA mice. ZJS also inhibited receptor activator of NF-κB ligand (RANKL)-induced osteoclast differentiation and the expression of MMP9 and cathepsin K *in vitro*. Mechanistically, ZJS blocked RANKL-induced p65 phosphorylation, nucleation, and inhibited the expression of downstream NFATc1 and c-Fos in bone marrow-derived macrophages (BMMs). Taken together, ZJS exerts a therapeutic effect on bone erosion in CIA mice by inhibiting RANKL/NF-κB-mediated osteoclast differentiation, which suggested that ZJS is a promising prescription for treating RA bone erosion.

## Introduction

Rheumatoid arthritis (RA) is a chronic autoimmune disease characterized by synovial inflammation, cartilage destruction, and bone erosion (Sparks, 2019). Approximately 1% of the world’s population suffers from RA, which occurs mainly in females (Smolen et al., 2016). Bone erosion appears early in the development of the disease and accompanies the entire progression of RA (Gravallese, 2002; Walsh and Gravallese, 2010). The main causes of bone erosion are synovitis, cytokine production, and osteoclast differentiation (Schett and Gravallese, 2012). NF-κB mediates osteoclast differentiation, which can be activated by receptor activator of NF-κB ligand (RANKL) in the inflammatory microenvironment (Jimi et al., 2004).

Currently, new targeted therapies and biomarkers have been identified for the diagnosis and treatment of RA bone erosion, while patients still develop unavoidable joint deformities (Burmester and Pope, 2017; Aletaha and Smolen, 2018). Disease-modifying anti-rheumatic drugs (DMARDs) and nonsteroidal anti-inflammatory drugs (NSAIDs) are common used in RA clinical trials, which fail to block the progression of bone erosion in RA patients (Orsolini et al., 2019). Biologic agents such as tocilizumab (IL-6R blockade) is effective in the reduction of bone erosion, but has limited effects on bone repair processes (Finzel et al., 2013). Denosumab, a monoclonal antibody against human RANKL, has induced membranous nephropathy in RA patients (Kimoto et al., 2020). Therefore, the effectiveness of these drugs is far from satisfactory, there is an urgent need to identify more efficacious drugs for treating RA bone erosion.

Traditional Chinese medicine has become popular as an alternative intervention to treat RA bone erosion. “Mi Yan Qi Zhen” reported that ZhiJingSan (ZJS)—a traditional Chinese prescription composed of scolopendra (dried body of *Scolopendra subspinipes mutilans L. Koch*, *scolopendridae*) and scorpion (dried body of *Buthus martensii Karsch*, *Buthus*)—can relieve pain, and improve rheumatism (Li, 2000). As ancient animal drugs in China, scolopendra and scorpion have attracted more attention because of their prominent effects in suppressing joint deformities (Qian and Wang, 2020). Previous studies found that a powder mixture of scolopendra and scorpion could improve joint swelling in collagen-induced arthritis (CIA) rats (Liu et al., 2012). Further studies demonstrated that polypeptides isolated from scolopendra and scorpion exhibit therapeutic effects against bone destruction in CIA rats (Park et al., 2018; Tanner et al., 2018).

Given that ZJS is closely correlated with RA therapy, the bone protection effect on RA bone erosion has yet to be demonstrated. This study was designed to investigate the inhibitory effects of ZJS on joint bone erosion in CIA mice and to explore its underlying mechanism. *In vivo*, the inhibitory effect of ZJS on arthritis severity, articular bone erosion, and the formation of osteoclasts as well as the expression of osteoclast marker proteins in CIA mice was evaluated. *In vitro*, the osteoclast differentiation model was used to evaluate the effect of ZJS on RANKL-mediated osteoclastogenesis and the key genes and proteins responsible for osteoclast differentiation. Mechanistically, the effect of ZJS on RANKL-mediated activation of NF-κB signaling pathway was clarified. Overall, our research showed that ZJS possesses the therapeutic potential in RA bone erosion.

## Materials and Methods

### Animals

DBA/1J female mice (6–8 weeks old, weighing 18–20 g) were purchased from Changzhou Cavins Laboratory Animal Co., Ltd. The mice were raised at a suitable temperature (18–29°C) with a 12 h light/dark cycle. Animal welfare and experimental procedures were performed strictly in accordance with the Animal Welfare Law of China and the Animal Ethics Committee of the Affiliated Hospital of Integrated Traditional Chinese and Western Medicine, Nanjing University of Chinese Medicine (AEWC-20200819-125).

### Chemicals and Reagents

Scorpion (batch number: 18080115) and Scolopendra (batch number: 20180601) were identified and provided by Jiangsu Province Hospital on the Integration of Chinese and Western Medicine (Nanjing, Jiangsu, China). Methotrexate (batch number: H31020644) was purchased from SPH Sine Pharmaceutical Laboratories Co., Ltd. (Shanghai, China). Bovine type II collagen and Freund’s adjuvant were purchased from Chondrex (Redmond, WA, United States). TRAP staining kits were purchased from Sigma-Aldrich (St. Louis, MO, United States). Antibodies against RANKL, OPG, cathepsin K, NFATc1, and c-Fos were purchased from Santa Cruz Biotechnology (Santa Cruz, CA, United States). Antibodies against MMP9, p65, and phosphorylated p65 were purchased from Cell Signaling Technology (Danvers, MA, United States). M-CSF was sourced from PeproTech Technology (Rocky Hill, NJ, United States). ELISA kits for IL-6, IL-10, IL-17, TNF-α were purchased from R&D Systems (Minneapolis, MN, United States) and ELISA kits for RANKL were purchased from Multi Sciences (Lianke) Biotech, Co., Ltd. (Hangzhou, China). ELISA kits for anti-bovine collagen II-specific antibodies (anti-bovine CII-specific Abs) were purchased from Chondrex (Redmond, WA, United States).

### Preparation of ZhiJingSan

To obtain ZJS for the *in vivo* experiments, the processed scorpion and scolopendra were crushed and passed through an 80-mesh sieve to form a fine powder, and further crushed using a cryogenic ball mill. Finally, the two powder was mixed with double distilled water at a mass ratio of 1:1 (the clinical dosage of ZJS was 1 g per day). *In vitro*, ZJS aqueous extract was prepared to obtain a freeze-dried powder, which was then dissolved in sterilized distilled water for the treatment of bone marrow-derived macrophages (BMMs).

### Induction of Collagen-Induced Arthritis and Drug Administration

DBA/1J female mice (6–8 weeks old) were randomly divided into four groups: normal (normal, *n* = 7), vehicle (vehicle, *n* = 7), methotrexate (MTX, *n* = 7), and ZhiJingSan group (ZJS, *n* = 7). The mice were immunized twice with bovine type II collagen, as previously reported (Courtenay et al., 1980) in the vehicle, methotrexate, and ZhiJingSan groups. For the first immunization, 100 μg of bovine collagen II dissolved in Freund’s complete adjuvant (CFA) was injected intradermally at the base of the tail of each mouse. On day 21, an immunization booster was administered in the form of 100 μg of bovine collagen II dissolved in Freund's incomplete adjuvant (IFA). On day 28, ZJS was intragastrically (i.g.) administered at a dose of 0.18 g/kg/day, and MTX was administered i.g. at a dose of 0.92 mg/kg twice a week. The dosage of ZJS and MTX was determined according to the clinical dosage in humans, calculated as follows: dose in mice equivalent experiment [g/kg] = [human dose (g)/body weight (60 kg)] × 11. The clinical dosage of ZJS was 1 g per person for every day, and the clinical dosage of MTX was 5 mg per time for twice a week. The normal and vehicle groups were administered an equal volume of deionized water. All the mice were treated for 30 days.

### Arthritis Assessment

After the booster immunization, the arthritic score, hind paw swelling, and body weight were measured every 3 days. The paw withdrawal threshold was detected in response to von Frey filaments (Chaplan et al., 1994). Arthritis severity was evaluated based on the arthritic score, which varies from 0 to 4 according to the following scale: 0, no signs; 1, detectable arthritis with some erythema; 2, significant redness and swelling; 3, severe redness and swelling from joint to digit; and 4, maximal swelling with arthrokleisis. All joints were scored separately, and the highest score obtained for each mouse was 16 (Honda et al., 2006).

### Hematoxylin-Eosin Staining and Micro-CT Scan

The limb joints of each mouse were fixed in a 4% paraformaldehyde solution, decalcified for 1 month using 10% EDTA, embedded in paraffin, sectioned, and stained with hematoxylin and eosin. The degree of histopathological damage was based on previously described criteria (Luo et al., 2013) and scored on a scale of 0–4 according to the degree of inflammatory cell infiltration, synovial hyperplasia. The fixed hind paws were placed in a centrifuge tube with physiological saline and scanned using a compact micro-CT scanner (SkyScan 1176, Bruker, Germany). Bone mineral density (BMD), bone surface/bone volume (BS/BV), bone volume fraction (BV/TV), trabecular thickness (Tb.Th), and trabecular separation (Tb.Sp) were detected to reflect bone mass, which were analyzed using the built-in analysis software.

### Enzyme-Linked Immunosorbent Assay

The sera of the mice were collected and stored at −80°C. The serum levels of IL-6, IL-10, TNF-α, IL-17, RANKL, and anti-bovine CII-specific Abs were measured using ELISA kits, according to the manufacturer’s instructions.

### Tartrate-Resistant Acid Phosphatase Staining and Immunohistochemistry

Sections of the ankle joint of each mouse were stained using a TRAP staining kit to identify osteoclasts. Multinucleated cells with more than three nuclei of TRAP-positive cells were considered osteoclasts. OPG, RANKL, and cathepsin K were immunolocalized by incubation with different primary antibodies. A light microscope was used for image processing, and the immunohistochemistry signals were quantified using ImageJ software.

### Cell Culture

Bone marrow mononuclear cells were separated from the tibias and femurs of 4–6 weeks-old C57BL/6 mice. Cells were cultured in α-MEM containing 10% FBS and 1% penicillin/streptomycin supplemented with 30 ng/ml M-CSF for 3 days. The adherent cells left at the bottom of the culture dish were considered BMMs.

### Cell Viability Assay

The ZJS water extract was used for the *in vitro* experiments after sterilization. Cell viability was measured using the MTT assay. BMMs (8 × 10^3^ cells/well) were inoculated in 96-well plates in triplicate and supplemented with 30 ng/ml M-CSF. After 12 h of culture, the cells were treated with different concentrations of ZJS (50–900 μg/ml) for 72 h. The optical density (OD) was measured at 570 nm.

### *In Vitro* Osteoclast Differentiation and Tartrate-Resistant Acid Phosphatase Staining

For the osteoclast differentiation, BMMs were inoculated in 96-well plates (8 × 10^3^ cells/well) and pretreated with or without ZJS (100, 150, 200, and 250 μg/ml) for 2 h. Then, the cells were cultured for 5 days, followed by stimulation with RANKL (100 ng/ml). The culture medium was replaced every other day. On the fifth day, the cells were fixed and stained with TRAP solution according to the manufacturer’s protocol. TRAP-positive cells with more than three nuclei were counted under a light microscope.

### RNA Extraction and Real-Time Quantitative PCR Assays

BMMs (5 × 10^5^ cells/well) were inoculated into 6-well plates, pretreated with or without ZJS (100, 150, and 200 μg/ml) for 2 h, and then stimulated with RANKL (100 ng/ml) for 48 h. Total RNA was extracted using TRIzol reagent according to the manufacturer’s instructions. A total of 1 μg of extracted RNA was reverse-transcribed to synthesize cDNA. cDNA was used as a template for RT-qPCR analysis using the SYBR Green qPCR Master Mix (Vazyme, Jiangsu, China). β-actin was used as the internal reference. The 2^−ΔΔCt^ method was used for the data analysis. The primer sequences used are shown in Table 1.

**TABLE 1:** Primers used for real-time qPCR.

Gene	Forward	Reverse
Murine *MMP9*	CTG​GAC​AGC​CAG​ACA​CTA​AAG	CTC​GCG​GCA​AGT​CTT​CAG​AG
Murine *cathepsin K*	GAA​GAA​GAC​TCA​CCA​GAA​GCA​G	TCC​AGG​TTA​TGG​GCA​GAG​ATT
Murine β*-actin*	AAC​AGT​CCG​CCT​AGA​AGC​AC	CGT​TGA​CAT​CCG​TAA​AGA​CC

### Western Blotting and Immunofluorescence

BMMs (5 × 10^5^ cells/well) were seeded in 6-well plates for 12 h. The cells were pretreated with or without ZJS (100, 150, and 200 μg/ml) for 2 h and then stimulated with 100 ng/ml RANKL for 48 h. The protein levels of NFATc1, c-Fos, MMP9, and cathepsin K were detected using western blotting. In addition, BMMs were treated with or without ZJS (100, 150, and 200 μg/ml) for 12 h, followed by stimulation with RANKL (100 ng/ml) for 1 h to detect the protein expression of p65 and p-p65. Data were analyzed using ImageJ software. Nucleation of p65 was detected by immunofluorescence staining. BMMs were pretreated with or without ZJS (200 μg/ml) for 12 h and then stimulated with 100 ng/ml RANKL for 1 h. Nucleation of p65 was detected using a laser scanning confocal microscope (FluoView Fvloi, Olympus, Japan).

### HPLC-Q-TOF-MS Analysis

ZJS powder (1 g) was dissolved in 80% methanol, and a solution was obtained by sonication for 30 min. Finally, the solution was analyzed using an HPLC-Q-TOF-MS system. Chromatographic separation was performed using an Agilent C18 column (3.0 mm × 100 mm, 2.7 μm; Agilent Technologies, Santa Clara, CA, United States) at 40°C. The mobile phase consisted of water containing 0.1% formic acid (A) and acetonitrile (B). The gradient program was set as: 0–0.01 min, 5% B; 0.01–25 min, 5–95% B; 25–27 min, 5% B; 27–30 min, 5% B. The mobile phase flow rate was 0.3 ml/min, and the sample injection volume was 2 μL. Electrospray ionization (ESI) in the positive ion mode was used for mass detection. The source parameters were set as follows: spray voltage, 4.5 kV; gas temperature, 550°C; pressure of nebulizer gas, 55 psi; full scan range, 50–1,000 m/z.

## Statistical Analysis

Data are expressed as mean ± standard error of the mean (SEM). One-way analysis of variance (ANOVA) and two-way ANOVA were used to evaluate intergroup variation. Differences among groups were assessed using Tukey’s multiple comparison test. Statistical significance was set at *p* < 0.05.

## Results

### ZhiJingSan Delayed the Onset of Arthritis and Ameliorated Arthritis Severity in Collagen-Induced Arthritis Mice

**FIGURE 1:**
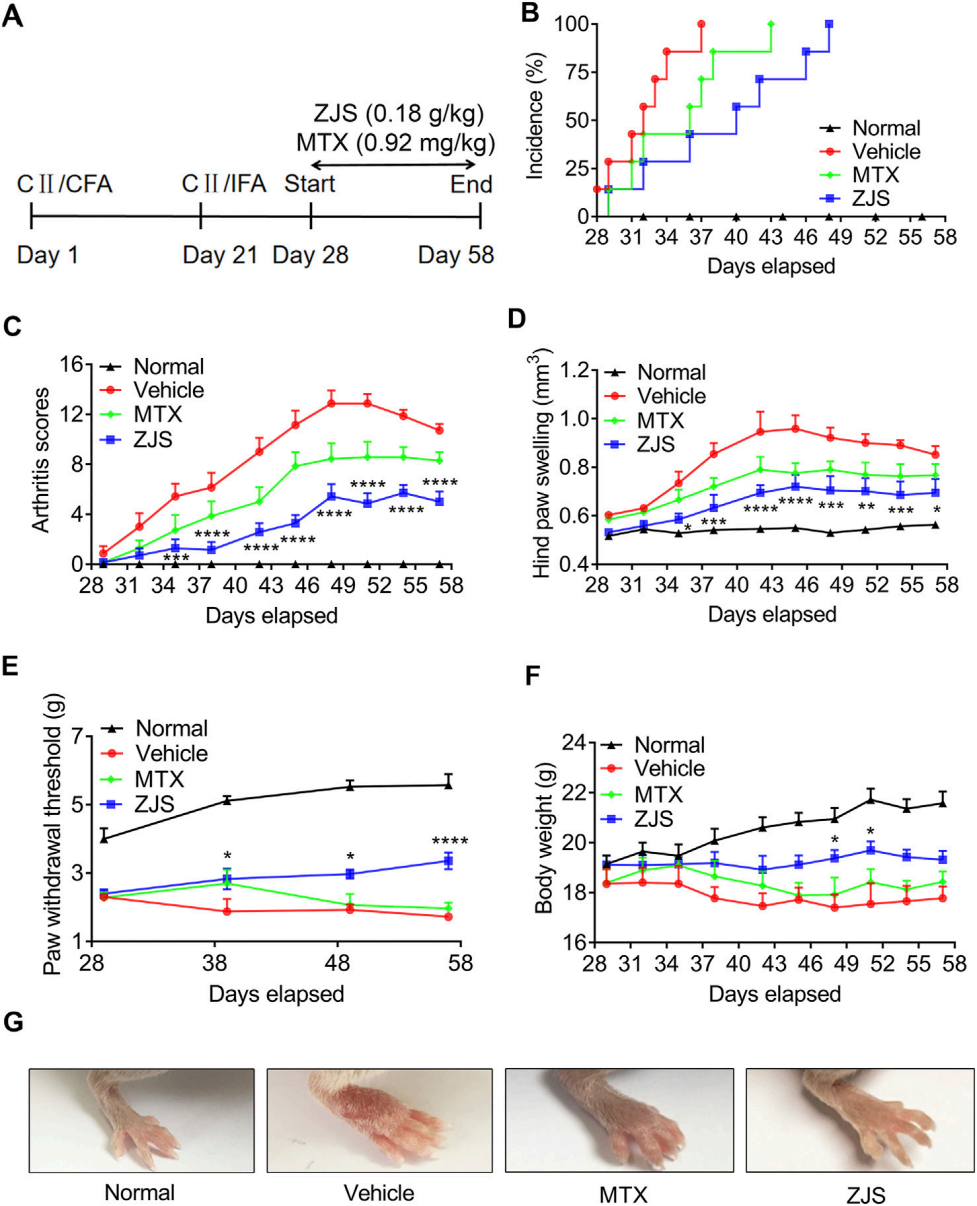
ZJS delayed the onset of arthritis and ameliorated arthritis severity in CIA mice. **(A)** Experimental scheme of the analysis of CIA mice. **(B)** Incidence of arthritis in each group was determined (*n* = 7). **(C)** Changes in the arthritis score were assessed in each group (*n* = 7). **(D)** Changes in the hind paw swelling were measured in each group (*n* = 7). **(E)** Changes in the paw withdrawal threshold were detected in each group using von Frey filaments (*n* = 7). **(F)** Changes in body weight were measured in each group (*n* = 7). **(G)** Representative images of the ankle joints of the mice taken on day 51 from the experiment in the indicated groups. Values are presented as the mean ± SEM. *p < 0.05, **p < 0.01, ***p < 0.005, ****p < 0.001, compared with the vehicle group (Two-way ANOVA test).

The component analysis of ZJS was shown in Table 2 and Supplementary Figure S1. To evaluate the therapeutic effect of ZJS on CIA mice, the onset of arthritis, the arthritic score, and hind paw swelling of CIA mice were determined, and the experimental scheme for the analysis of CIA mice was shown in Figure 1A. All the vehicle-treated mice showed symptoms of arthritis on day 37, and ZJS treatment effectively delayed the onset of arthritis in CIA mice (Figure 1B). The arthritic score of the vehicle group peaked after booster immunization for 4 weeks, ZJS treatment decreased the arthritic score and hind paw swelling compared to those of the vehicle group from day 35 to the end of treatment (Figures 1C,D,G). The paw withdrawal threshold was used to characterize pain sensitization in the ankle joints of the CIA mice. As arthritis progressed, ZJS treatment significantly reduced the mechanical allodynia in CIA mice on days 39, 49, and 57 (Figure 1E). Additionally, weight loss was significant in the vehicle-treated and MTX-treated mice, and ZJS alleviated weight loss on days 48 and 51 compared with the vehicle group (Figure 1F).

**TABLE 2:** The mass information of the compounds identified in ZJS using HPLC-Q-TOF-MS.

No.	Rt (min)	Identification	Formula	m/z	Error (ppm)
1	0.66	L (+)-arginine	C_6_H_14_N_4_O_2_	[M + H]^+^ 175.1195	3
2	0.68	Valine	C_5_H_11_NO_2_	[M + H]^+^ 140.0684	1.3
3	0.75	2,6-Dihydroxypurine	C_5_H_4_N_4_O_2_	[M + COOH]^+^ 197.0313	3.7
4	0.97	Uracil	C_4_H_4_N_2_O_2_	[M + H]^+^ 113.0348	1.9
5	1.13	Methyl palmitate	C_17_H_34_O_2_	[M + H]^+^ 271.2631	−0.1
6	2.36	3,8-Dihydroxyquinoline	C_9_H_7_NO_2_	[M + H]^+^ 162.0550	0
7	3.93	2-Heptanone	C_7_H_14_O	[M + H]^+^ 115.1119	1.1
8	4.74	3-Indoleacetamide	C_10_H_10_N_2_O	[M + H]^+^ 175.0868	1.4
9	5.64	L-Aspartic acid	C_4_H_7_NO_4_	[M + H]^+^ 134.0452	2.7
10	6.08	L-Threonine	C_4_H_9_NO_3_	[M + H]^+^ 120.0657	1.7
11	6.83	Salbutamol	C_13_H_21_NO_3_	[M + H]^+^ 240.1595	0.5
12	7.5	6-Methyl-5-hepten-2-one	C_8_H_14_O	[M + H]^+^ 127.1121	2.4
13	9.95	6-Hydroxypurine	C_5_H_4_N_4_O	[M + H]^+^ 159.0275	−1.6
14	10.78	L-Isoleucine	C_6_H_13_NO_2_	[M + H]^+^ 132.1021	1.1
15	11.07	Guanosine	C_10_H_13_N_5_O_5_	[M + H]^+^ 284.1004	4.9
16	11.11	Phenylethylamine	C_8_H_11_N	[M + COOH]^+^ 197.0313	3.1
17	11.37	2-Nonanone	C_9_H_18_O	[M + H]^+^ 143.1428	−1.8
18	14.14	β-Farnesene	C_15_H_24_	[M + COOH]^+^ 249.1857	3.2
19	14.66	Linolenic acid	C_18_H_30_O_2_	[M + COOH]^+^ 323.2224	2.2

### ZhiJingSan Inhibited Cartilage Damage, Anti-Bovine CII-Specific Abs Production, and Inflammatory Cytokines in Collagen-Induced Arthritis Mice

**FIGURE 2:**
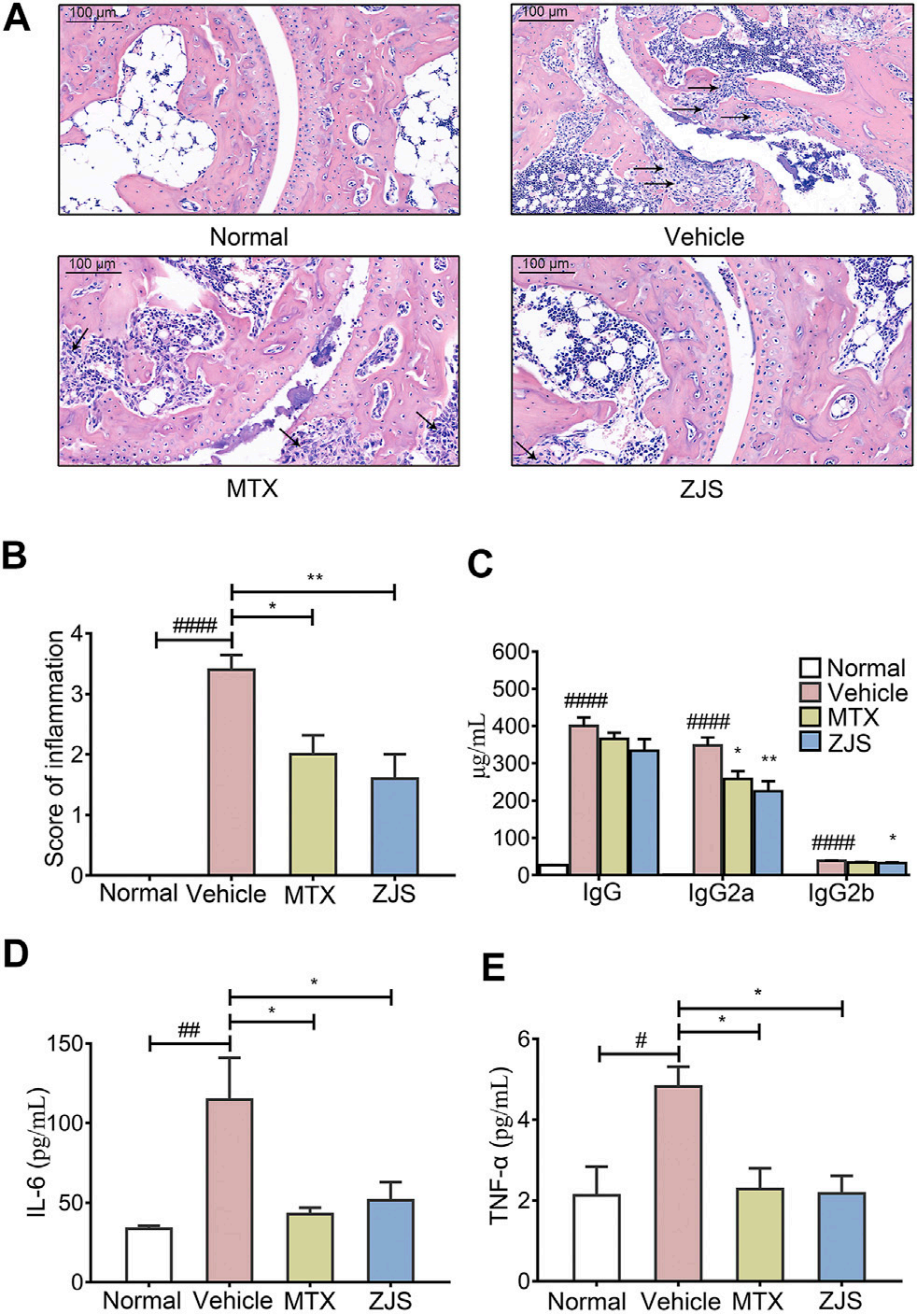
ZJS inhibited cartilage damage, anti-CII specific Abs production, and inflammatory cytokines in CIA mice. **(A)** Representative pathological images of ankle joints stained with HE, the arrows indicated the synovial cell hyperplasia in the pathological images of ankle joints (scale bar = 100 μm). **(B)** The pathological assessment of ankle joints in the indicated groups (*n* = 5). **(C)** The levels of anti-bovine CII-specific IgG and isotype-specific IgG2a and IgG2b antibodies in mouse serum (*n* = 5). **(D,E)** The levels of pro-inflammatory cytokines (IL-6 and TNF-α) in mouse serum (*n* = 5). Values are presented as the mean ± SEM. ^#^ *p* < 0.05, ^##^ *p* < 0.01, ^####^ *p* < 0.001, compared with the normal group. *p < 0.05, **p < 0.01, compared with the vehicle group (One-way ANOVA test).

As noted, ZJS ameliorated arthritis severity in CIA mice, the effects of ZJS on pathological changes in the ankle joints of CIA mice were then verified. ZJS treatment significantly decreased levels of inflammatory cell infiltration, synovial hyperplasia, and severe cartilage erosion in the ankle joints of CIA mice compared with the vehicle group (Figure 2A). Meanwhile, the inflammation scores in ZJS-treated mice were significantly lower than those in vehicle-treated mice (Figure 2B). As shown in Figure 2C, the serum levels of anti-bovine CII-specific IgG, IgG2a, and IgG2b antibodies were significantly lower in ZJS-treated mice than in vehicle-treated mice. Moreover, the serum levels of inflammatory cytokines IL-6 and TNF-α were significantly decreased in the ZJS group (Figures 2D,E). While the serum levels of IL-10 and IL-17 in ZJS-treated CIA mice exhibited no significant changes compared with vehicle-treated mice (Supplementary Figure S2).

### ZhiJingSan Inhibited Joint Bone Erosion in Collagen-Induced Arthritis Mice

**FIGURE 3:**
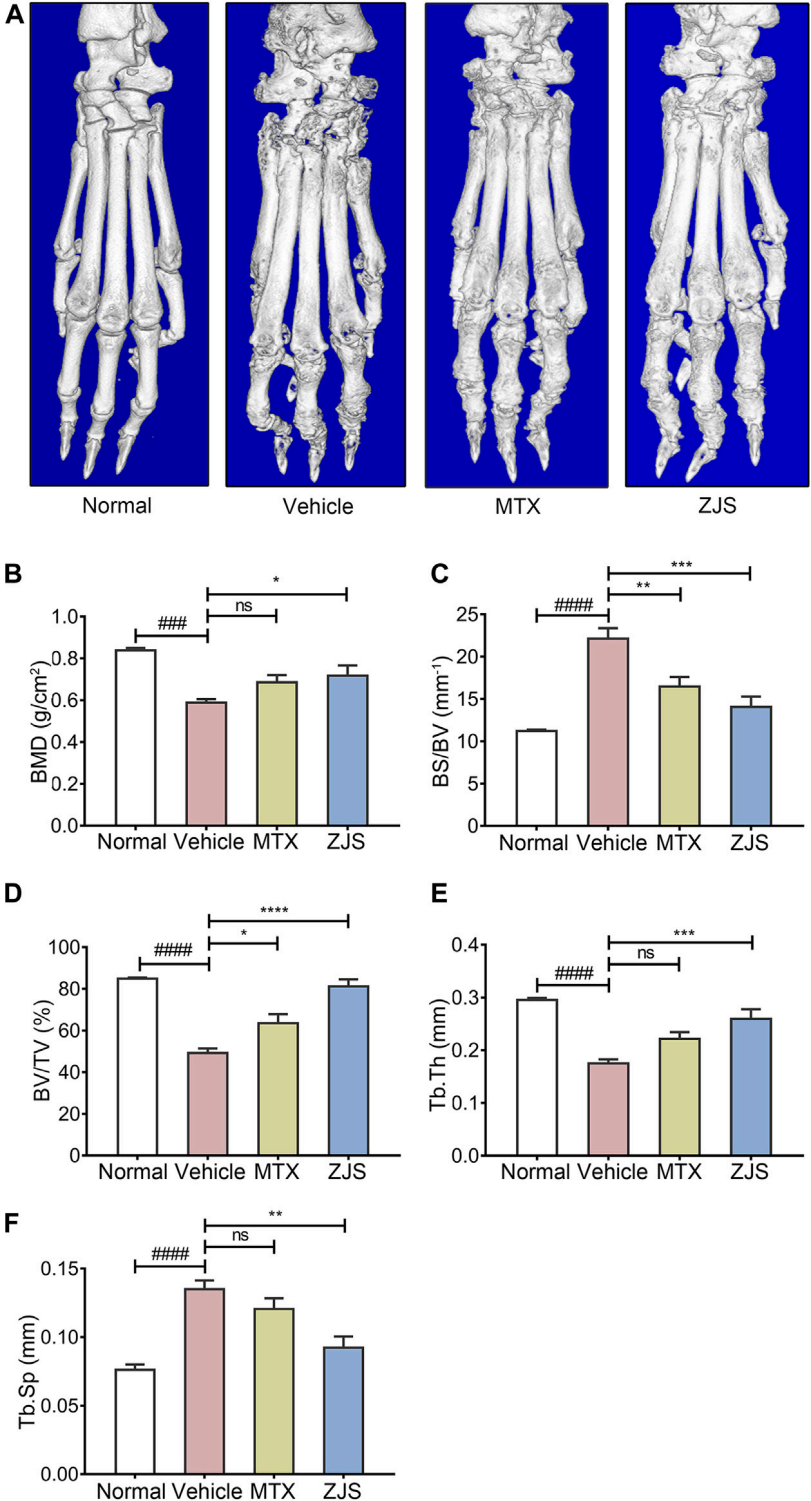
ZJS suppressed joint bone erosion in CIA mice. **(A)** Representative three-dimensional renditions of hind paws from mice in the indicated groups, which were obtained using micro-CT. **(B)** Bone mineral density (BMD) of the ankle joints in the indicated groups (*n* = 5). **(C)** The ratio of bone surface to bone volume (BS/BV) of the ankle joints in the indicated groups (*n* = 5). **(D)** Bone volume fraction (BV/TV) of the ankle joints in the indicated groups (*n* = 5). **(E)** The bone trabecular thickness (Tb.Th) of the ankle joints in the indicated groups (*n* = 5). **(F)** Bone trabecular separation (Tb.Sp) of the ankle joints in the indicated groups (*n* = 5). Values are presented as the mean ± SEM. ns: no significance. ^###^ *p* < 0.005, ^####^ *p* < 0.001, compared to the normal group. *p < 0.05, **p < 0.01, ***p < 0.005, ****p < 0.001, compared with the vehicle group (One-way ANOVA test).

To validate the effect of ZJS on joint bone erosion in CIA mice, the hind paws of the mice from all groups were examined using micro-CT. As shown in Figure 3A, micro-CT images showed that the surface of the ankle joints in normal mice was smooth, while the rough bone surface, severe bone resorption, joint bone destruction, and joint space enlargement were observed in the ankle joints of the vehicle-treated mice. ZJS treatment led to a remarkable reduction in joint destruction compared to that in vehicle-treated mice. Bone microfracture parameters, including BMD, BV/TV, Tb.Sp, Tb.Th, and BS/BV were measured to quantify the extent of joint destruction in the different groups. The ZJS group markedly increased BMD, BV/TV, and Tb.Th values, and reduced BS/BV and Tb.Sp values compared to the vehicle group (Figures 3B–F).

### ZhiJingSan Reduced the Number of Osteoclasts in the Ankle Joints of Collagen-Induced Arthritis Mice

**FIGURE 4:**
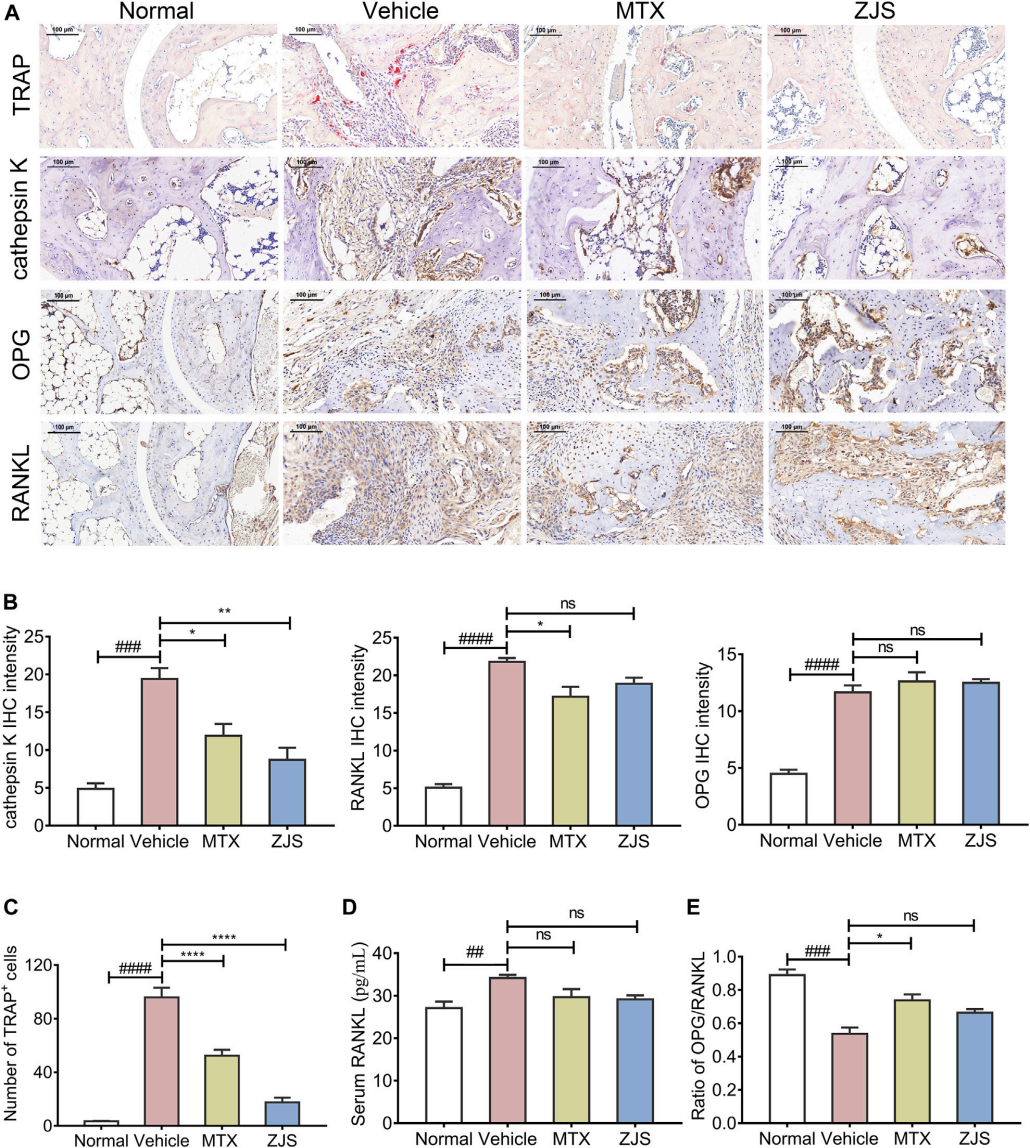
ZJS decreased the number of osteoclasts and the expression of osteoclast-related proteins in the ankle joints of CIA mice. **(A)** Representative immunohistochemical staining images of TRAP, cathepsin K, OPG, and RANKL in the ankle joints of mice in the indicated groups (scales bars = 100 μm). **(B)** The quantification of cathepsin K, RANKL, and OPG expression in the ankle joints of mice in the indicated groups (*n* = 3). **(C)** The number of TRAP-positive cells in the ankle joints in the indicated groups (*n* = 5). **(D)** The content of RANKL in the serum of mice in the indicated groups (*n* = 5). **(E)** Ratio of the IHC intensity of OPG and RANKL in the ankle joints in the indicated groups (*n* = 3). Values are presented as the mean ± SEM. ns: no significance. ^##^ *p* < 0.01, ^###^ *p* < 0.005, ^####^ *p* < 0.001, compared with the normal group. *p < 0.05, **p < 0.01, ****p < 0.001, compared with the vehicle group (One-way ANOVA test).

Osteoclasts in the ankle joints of the mice were characterized by TRAP staining. Compared with the vehicle-treated mice, ZJS significantly reduced the number of osteoclasts in the ankle joints of CIA mice (Figures 4A,C). OPG, RANKL, and cathepsin K were detected in the ankle joints to evaluate the effect of ZJS on the production of osteoclasts. As shown in Figures 4A,B, the expression level of cathepsin K, which is a marker of osteoclastogenesis, was significantly reduced in the ankle joints of ZJS-treated mice compared with vehicle-treated mice. However, there were no significant changes in the expression of OPG, RANKL and ratio of OPG/RANKL in the ankle joints (Figures 4A,B,E) or in the serum level of RANKL in ZJS-treated mice compared with vehicle-treated mice (Figure 4D).

### ZhiJingSan Inhibited Receptor Activator of NF-κB Ligand-Induced Osteoclast Differentiation *In Vitro*

**FIGURE 5:**
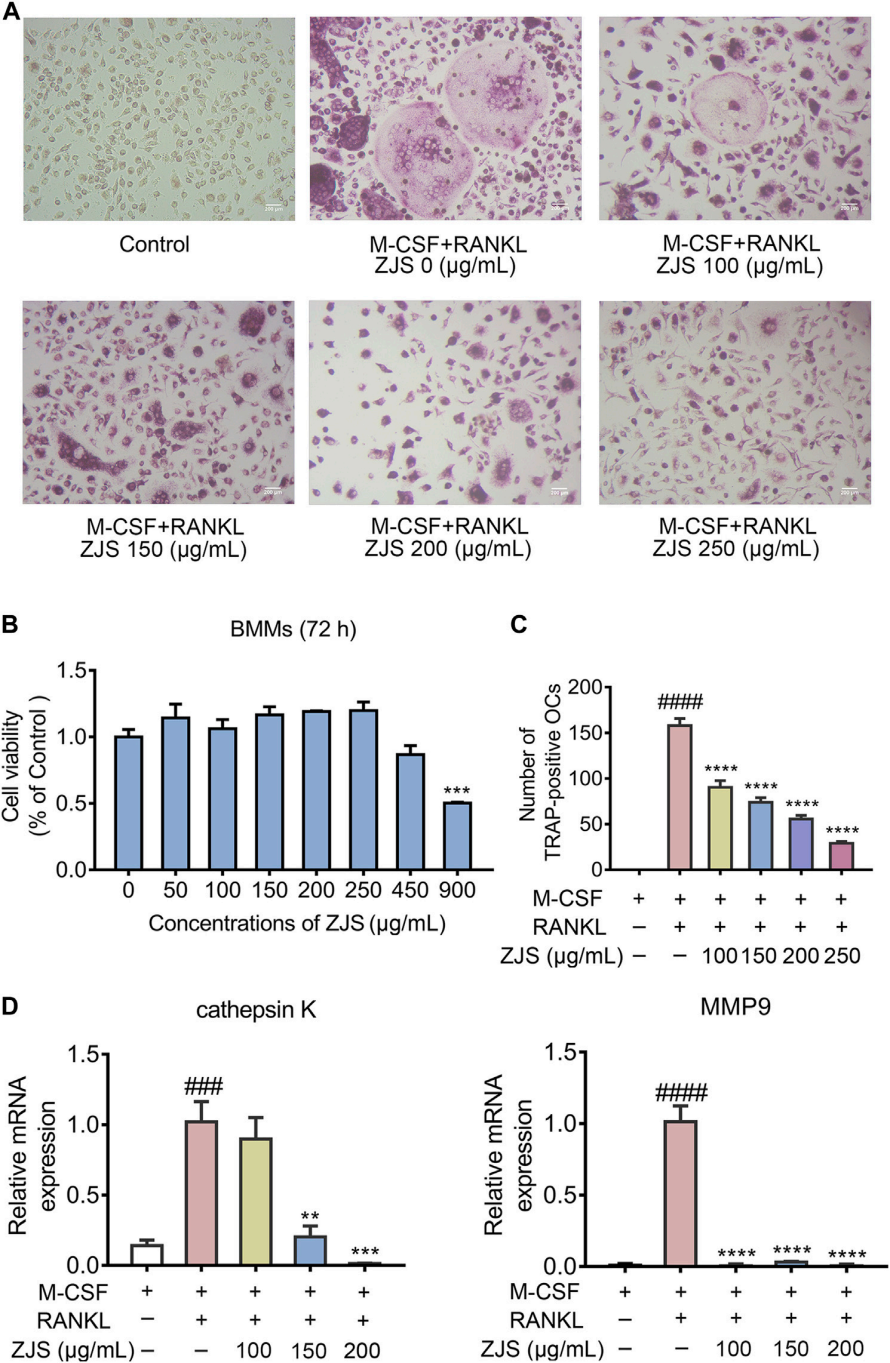
ZJS inhibited the differentiation of osteoclasts and the mRNA expression of osteoclast-related genes *in vitro.* **(A)** Representative images of TRAP-positive cells in BMMs treated with the indicated concentrations of ZJS followed by stimulation with RANKL (scale bars = 200 μm). **(B)** Cell viability was evaluated using MTT assay (*n* = 3). **(C)** The number of TRAP-positive multinucleate osteoclasts treated with indicated concentrations of ZJS followed by stimulation with RANKL (*n* = 3). **(D)** The mRNA expression levels of cathepsin K and MMP9 in BMMs in the indicated groups were determined by real time qPCR analysis, β-actin was used as the internal reference (*n* = 3). Values are presented as the mean ± SEM. ^*###*^ *p* < 0.005, ^*####*^ *p* < 0.001, compared with the RANKL-untreated group. **p < 0.01, ***p < 0.005, ****p < 0.001, compared with the RANKL + ZJS-untreated group (One-way ANOVA test).

ZJS decreased the number of osteoclasts in the ankle joints of CIA mice, but did not affect the level of RANKL in sera and the ratio of OPG/RANKL expressed in joints. We then explored whether ZJS could inhibit osteoclast differentiation *in vitro*. The data showed that BMMs viability was not affected by ZJS up to a concentration of 250 μg/ml (Figure 5B). TRAP-positive cells (>3 nuclei) were observed upon RANKL stimulation, ZJS (100, 150, 200, and 250 μg/ml) treatment significantly decreased the number of TRAP-positive osteoclasts in a dose-dependent manner (Figures 5A,C). We next detected the effect of ZJS on the mRNA expression levels of osteoclast-specific genes (cathepsin K and MMP9). The upregulation of cathepsin K and MMP9 genes expression was observed in BMMs induced by RANKL, which was significantly reduced upon ZJS treatment for 48 h (Figure 5D). Taken together, these results further strengthened the hypothesis that ZJS effectively inhibited osteoclast formation.

### ZhiJingSan Suppressed NF-κB Signaling Pathway Induced by Receptor Activator of NF-κB Ligand

**FIGURE 6:**
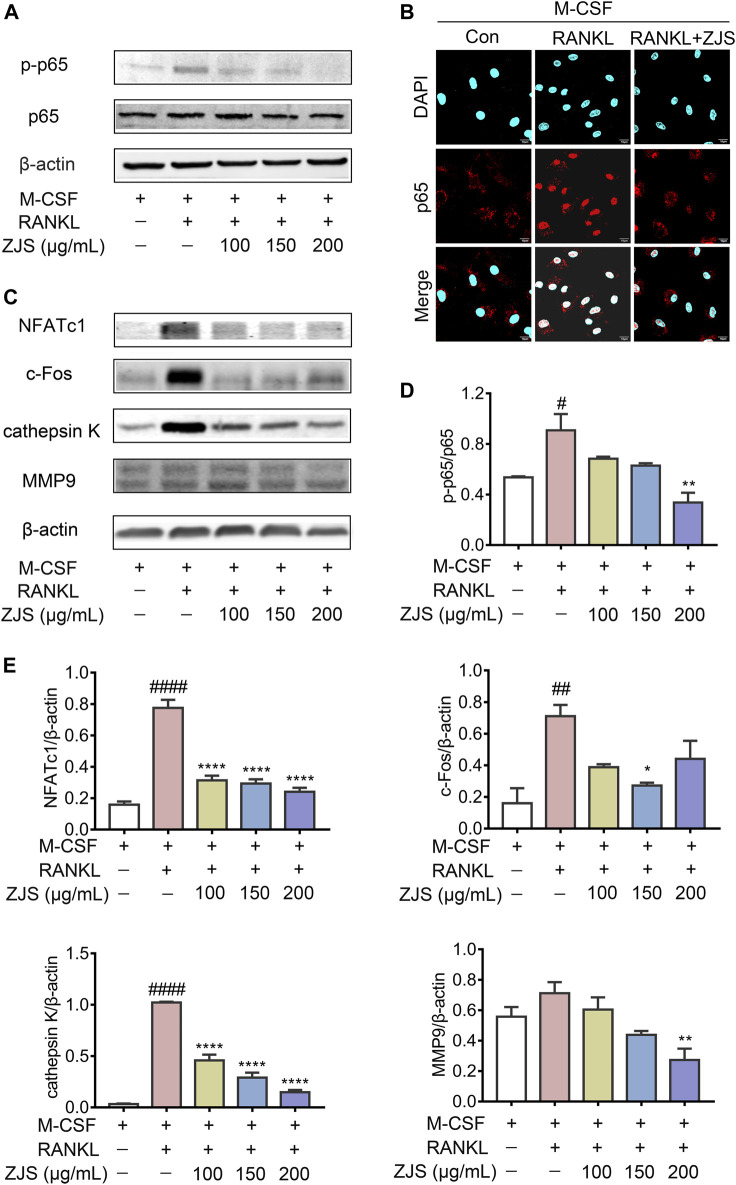
ZJS suppressed the RANKL-induced NF-κB signaling pathway. **(A)** Representative immunoblots of p65 and p-p65 in BMMs were performed by western blotting, β-actin was used as the internal reference protein. **(B)** Representative immunofluorescence images of p65 in the nucleus of BMMs in the indicated groups (scale bars = 10 μm). **(C)** Representative immunoblots of NFATc1, c-Fos, cathepsin K, and MMP9 in BMMs were performed by western blotting, β-actin was used as the internal reference protein. **(D)** Western blot analysis of p-p65 in BMMs in the indicated groups (*n* = 3). **(E)** Western blot analysis of NFATc1, c-Fos, cathepsin K, and MMP9 in BMMs in the indicated groups (*n* = 3). Values are presented as the mean ± SEM. ^*#*^ *p* < 0.05, ^*##*^ *p* < 0.01, ^*####*^ *p* < 0.001, compared with the RANKL-untreated group. *p < 0.05, **p < 0.01, ****p < 0.001, compared with the RANKL + ZJS-untreated group (One-way ANOVA test).

The NF-κB signaling pathway plays a critical role in mediating RANKL-induced osteoclast differentiation. We further explored the effect of ZJS on the RANKL-induced NF-κB signaling pathway in BMMs. The expression level of p-p65 was significantly increased by RANKL stimulation, which was inhibited by ZJS treatment (Figures 6A,D). Then we verified whether ZJS blocked the nucleation of p65. As shown in Figure 6B, p65 translocated into the nuclei after RANKL stimulation, and ZJS treatment inhibited the nucleation of p65. Moreover, ZJS significantly inhibited the increased protein level of MMP9, cathepsin K, c-Fos, and NFATc1 upon RANKL stimulation (Figures 6C,E). These data suggested that inhibition of the RANKL-induced NF-κB signaling pathway by ZJS was involved in the suppression of osteoclast differentiation.

## Discussion

RA treatment requires not only inflammation control, but also the inhibition of joint destruction and bone erosion (Guo et al., 2018). Bone erosion is a typical hallmark of RA (Scott et al., 2000; Welsing et al., 2001). Concomitantly with the development of bone loss, the main anti-inflammatory therapeutic agents showed poor protective benefits in repairing reduced bone mass (Dimitroulas et al., 2013). Therefore, it is necessary to develop novel therapeutic drugs for bone erosion. Chinese classical prescription has been an increasingly important source of drug treatment for RA. ZJS provides a great support in RA therapy, and shows the protection against joint deformities (Qian and Wang, 2020), while the therapeutic effects of ZJS on RA bone erosion is still unknown.

In this study, a CIA mouse model was used to investigate the effects of ZJS on RA inflammation and bone erosion. ZJS delayed the onset of arthritis and reduced the arthritic score. Meanwhile, ZJS decreased the serum levels of anti-bovine CII-specific Abs, IL-6, and TNF-α, which is consistent with previous reports (Liu et al., 2012). The results also showed that ZJS reduced the mechanical allodynia, thus relieving the joint pain in CIA mice. MTX, a positive drug in this study, could suppress dihydrofolate reductase and DNA synthesis, has proven to be a first-line anti-rheumatic agent, which inhibits the progression of RA mainly by inflammation control (Wang et al., 2018). However, the results indicated that ZJS was more effective in inhibiting inflammation than MTX. Additionally, there were no obvious benefits in regulating weight loss and mechanical allodynia after MTX treatment.

Furthermore, ZJS significantly reduced joint synovitis, articular cartilage, and bone damage in CIA mice. There were further evident improvements in bone indices (including BMD, BS/BV, BV/TV, Tb.Th, and Tb.Sp) after ZJS treatment. Bone erosion in RA-affected joints arises from the activation of osteoclasts by inflammatory processes (Adamopoulos and Mellins, 2015). Osteoclasts, which are derived from the monocyte lineage, are the primary bone resorptive cells and play an essential role in bone loss (Park-Min, 2018; Park-Min, 2019). Therefore, the purpose of treating RA bone erosion can be achieved by inhibiting osteoclast differentiation. The study then demonstrated that ZJS decreased the number of osteoclasts and the production of cathepsin K in the ankle joints of CIA mice. It is well known that osteoclasts resorb bone in the body by synthesizing cathepsin K, matrix metalloproteinases (MMPs), and TRAP (Teitelbaum and Ross, 2003). These results revealed that ZJS suppressed osteoclast differentiation *in vivo*.

RANKL is the most essential regulator capable of governing the processes of osteoclastogenesis (Park et al., 2017). OPG, a natural receptor inhibitor of RANKL, counteracts the overactivation of RANKL in osteoclast formation and maintains bone homeostasis (Theoleyre et al., 2004). The OPG/RANKL ratio plays an important role in regulating RA bone erosion. It is worth noting that ZJS had no effect on the level of RANKL in serum of CIA mice, and the ratio of OPG/RANKL expressed in the ankle joints, which suggested that the inhibition of osteoclast differentiation by ZJS may occur via the suppression of RANKL-mediated downstream signaling pathways.

Based on the *in vivo* results, *in vitro* experiments were conducted to explore the underlying mechanism of ZJS-mediated inhibition of osteoclast differentiation. BMMs were treated with RANKL to induce osteoclast differentiation. ZJS inhibited RANKL-induced osteoclastogenesis and decreased the production of osteoclast marker genes and proteins (cathepsin K and MMP9), which was in line with the *in vivo* study.

Activation of the NF-κB signaling pathway has been proved to be crucial in osteoclastogenesis, which can be induced by RANKL (Novack, 2011; Kikuta and Ishii, 2013). NF-κB signaling activation also leads to T cell activation and mediates an inflammatory response, resulting in bone loss direct by the abnormal activation of osteoclasts. (Jimi and Ghosh, 2005; Brown et al., 2008). The blocking of p65 nuclear translocation and the inhibition of p65 phosphorylation have been shown to suppress the pathogenesis of RA (Kang et al., 2018). In the present study, ZJS treatment inhibited the phosphorylation and nucleation of p65, indicating that the NF-κB signaling pathway is involved in the suppression of osteoclast differentiation by ZJS. The expression levels of IL-6 and TNF-α, which are immune response products of the NF-κB signaling pathway, were also decreased in the serum of ZJS-treated mice. Moreover, ZJS decreased the protein expression levels of c-Fos and NFATc1, which are transcription factors regulated by the NF-κB signaling pathway that can regulate osteoclast differentiation and osteoclast-specific gene expression (Grigoriadis et al., 1994; Wagner and Eferl, 2005). All these results illustrated that ZJS profoundly inhibited RANKL-induced osteoclast differentiation by downregulating the NF-κB signaling pathway.

It is necessary to analyze the monomer components of ZJS to better understand its therapeutic effects. A total of 19 compounds were preliminarily identified in ZJS (Table 2 and Supplementary Figure S1). These compounds included free amino acids, nucleotides, lipids, amines, and heterocyclic compounds, which is consistent with previous research results (Evans et al., 2020). Linolenic acid which we identified in ZJS is known as an n-3 fatty acid, and is considered essential for human health. Recent studies showed that linolenic acid inhibited RANKL induced osteoclast differentiation and suppressed inflammatory bone loss via modulating NF-κB-iNOS signaling pathway (Song et al., 2017). Salbutamol, as a β2-AR selective agonist, is proved to be a potent suppressor of CIA mouse model by suppressing the immunoinflammatory response (Malfait et al., 1999), and ameliorated joint inflammation in adjuvant-induced arthritic rats (Wu et al., 2016). Methyl palmitate significantly suppressed the expression of inflammatory cytokines (IL-1β and TNF-α) in adjuvant-induced arthritic rats, and exerted its anti-inflammatory effects in the treatment of RA (Abdel Jaleel et al., 2021). Whether these compounds are also involved in the regulation of ZJS in the treatment of RA bone erosion and the inhibition of osteoclast differentiation remains to be further studied. Additionally, the monomer compounds in ZJS may not be identified accurately in our current research. The identification of new monomer compounds in ZJS deserves further research.

In conclusion, this study demonstrates that ZJS as a traditional Chinese animal prescription, improved joint bone destruction of CIA mice and the protective effect was attributed to its inhibitory against RANKL/NF-κB-mediated osteoclast differentiation, suggesting that ZJS could be a potential prescription for RA bone erosion treatment.

## Data Availability

The raw data supporting the conclusion of this article will be made available by the authors, without undue reservation.
