# Supplementary material for: ZhiJingSan Inhibits Osteoclastogenesis via Regulating RANKL/NF-κB Signaling Pathway and Ameliorates Bone Erosion in Collagen-Induced Mouse Arthritis
Source: Front Pharmacol. 2021 May 28;12:693777. doi: 10.3389/fphar.2021.693777 (PMC8193094; doi:10.3389/fphar.2021.693777)
Supplement: Supplementary file 1 [file Presentation1.PPTX]

## Slide 1
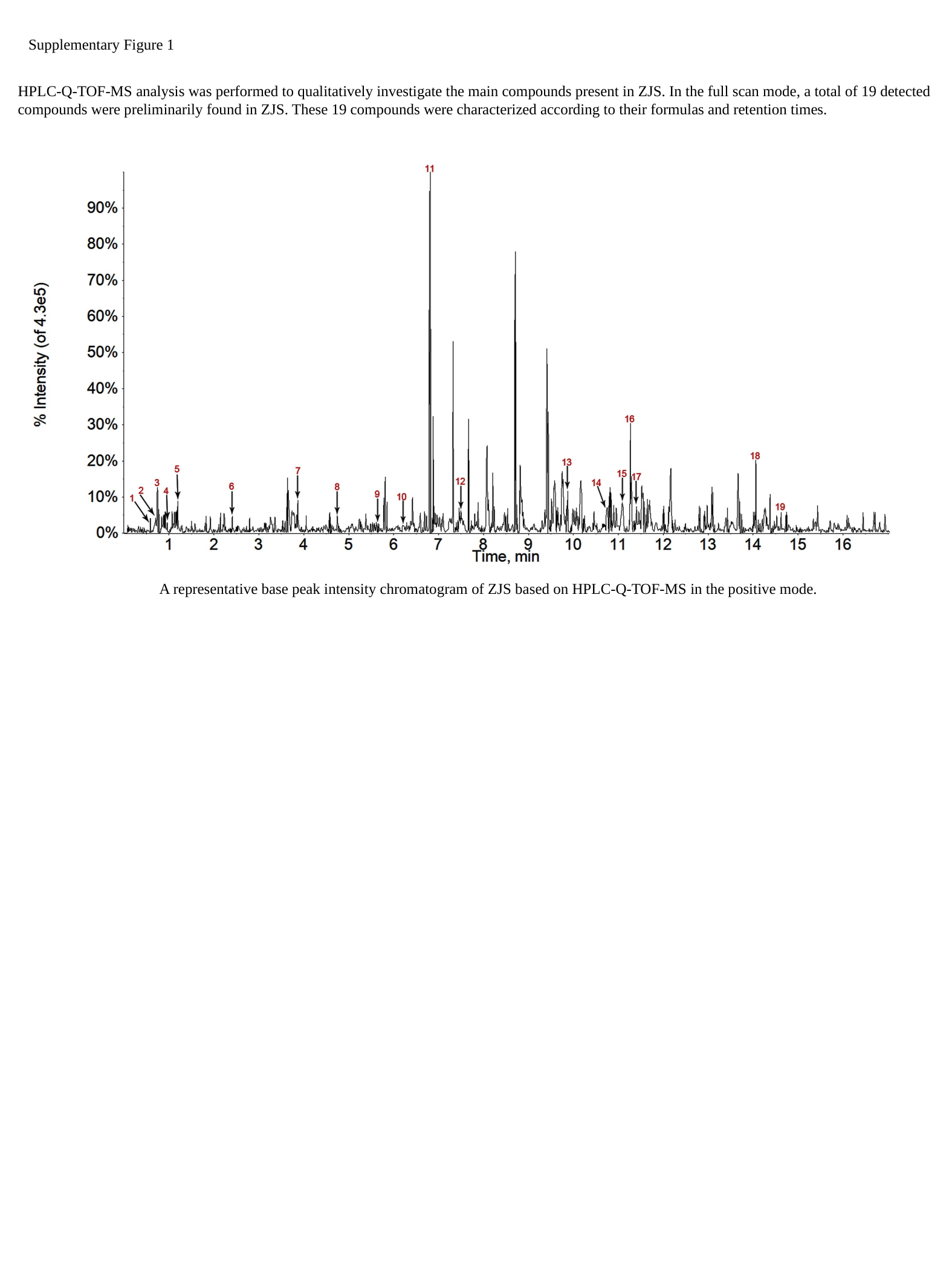

Supplementary Figure 1
HPLC-Q-TOF-MS analysis was performed to qualitatively investigate the main compounds present in ZJS. In the full scan mode, a total of 19 detected compounds were preliminarily found in ZJS. These 19 compounds were characterized according to their formulas and retention times.
A representative base peak intensity chromatogram of ZJS based on HPLC-Q-TOF-MS in the positive mode.

## Slide 2
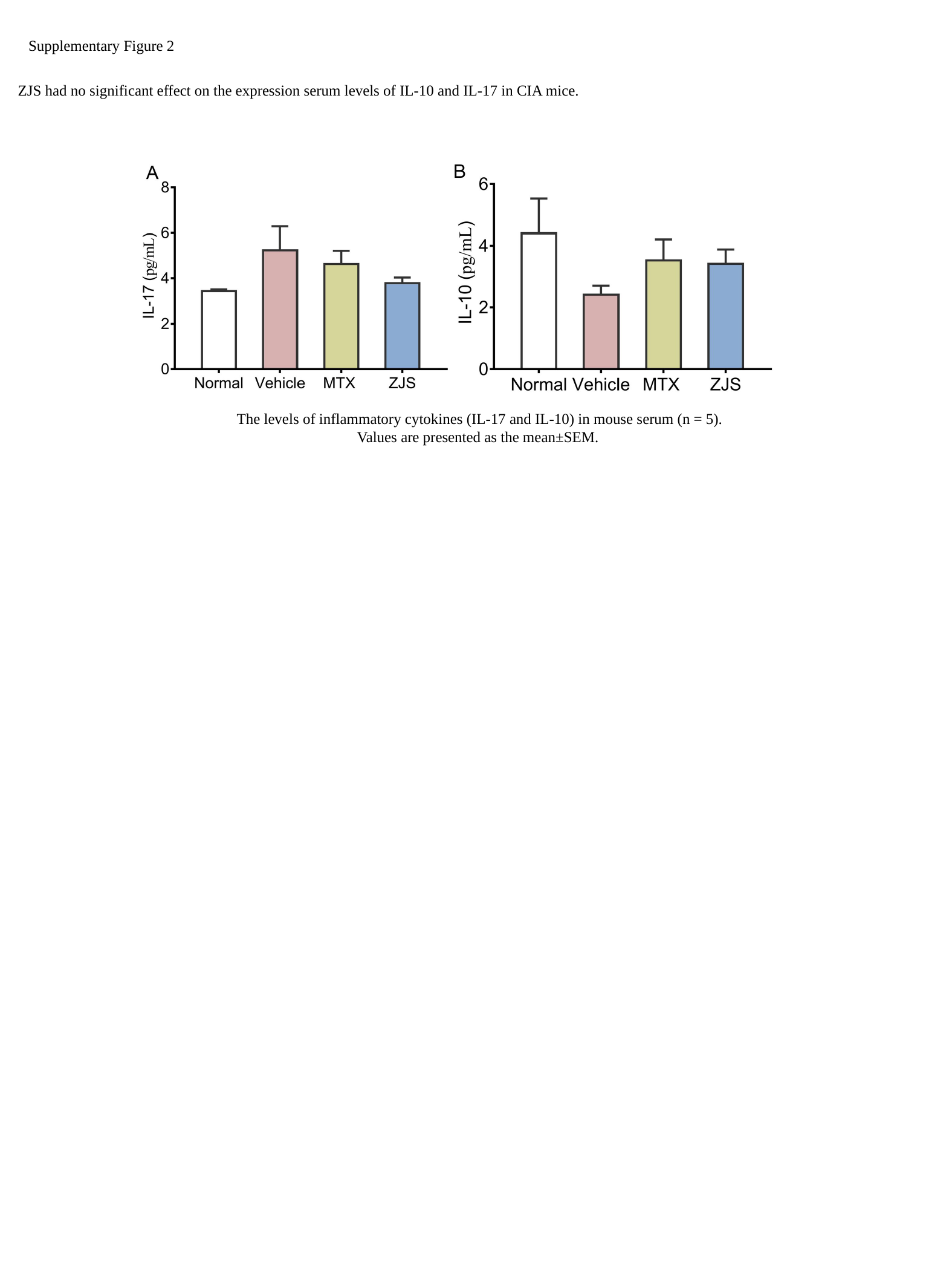

Supplementary Figure 2
ZJS had no significant effect on the expression serum levels of IL-10 and IL-17 in CIA mice.
The levels of inflammatory cytokines (IL-17 and IL-10) in mouse serum (n = 5).
Values are presented as the mean±SEM.
